# Coagulation Matters: ATIII‐Enriched Biomolecular Corona Enhances the Hemocompatibility of PEG Nanoparticles

**DOI:** 10.1002/adhm.202501431

**Published:** 2025-06-30

**Authors:** Vaidehi Londhe, Manfred F. Maitz, Triantafyllos Chavakis, Carsten Werner, Alessia C. G. Weiss, Quinn A. Besford

**Affiliations:** ^1^ Leibniz‐Institut für Polymerforschung Dresden e.V. Hohe Str. 6 01069 Dresden Germany; ^2^ Institute for Clinical Chemistry and Laboratory Medicine University Hospital Fetscherstr. 74 01307 Dresden Germany; ^3^ Faculty of Medicine TU Dresden Fiedlerstraße 27 01307 Dresden Germany

**Keywords:** anticoagulant, ATIII, biomolecular corona, low‐fouling, stealth

## Abstract

Nanoparticles in physiological environments acquire a biomolecular corona that defines their biological identity, mediating immune system recognition and accelerating blood clearance of the nanoparticles. Typically, low‐fouling materials are chosen to minimize protein adsorption and thereby immune system responses, contributing to stealth in blood. However, absolute prevention of the biomolecular corona remains tantalizingly out of reach. Herein, it is proposed to leverage the biomolecular corona rather than preventing its formation, in order to overcome immune responses toward nanoparticles. Low‐fouling and stealthy poly(ethylene glycol)(PEG) nanoparticles are used, with a functional biomolecular corona enriched with anticoagulant heparin‐antithrombin III (HEP‐ATIII) complexes that can mitigate undesirable immune responses. Through immune response evaluations and proteomic analyses are used to ascertain the low‐fouling, stealthy character of the nanoparticles, similar to that of PEG nanoparticles. However, PEG nanoparticles alone induce coagulation responses in human blood, which are mitigated by pre‐enrichment of the biomolecular corona with HEP‐ATIII complexes. This shows that coagulation is another factor to be considered in the design of materials for nanomedicine and that the low‐fouling and stealthy properties do not directly translate to hemocompatibility. These findings highlight the potential of biomolecular corona engineering to address key challenges in the field, toward developing safer, efficacious therapeutic nanomaterials.

## Graphical abstract

Engineering of a functional protein corona with a synergistic heparin‐ATIII complex significantly improves hemocompatibility of PEG nanoparticles. Through deliberate engineering of the protein corona, a 4‐fold reduction in coagulation activation (F1 + 2) without inducing adverse thromboinflammatory reactions (CD11b, C5a) is achieved without disrupting the inherent stealth and low‐fouling characteristics of PEG nanoparticles.

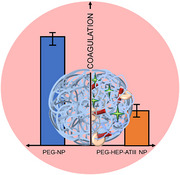

## 1 Introduction

A mechanistic understanding of the biological identity of nanoparticles (NPs) is crucial for their success as therapeutic carriers, where the biological identity is dictated by the formation of the biomolecular corona from biological fluids. The biomolecular corona is a layer of proteins, along with other biomolecules, that evolves on a NP's surface upon introduction to the complex biological milieu. It forms the nano‐bio interface and controls the downstream NP‐immune system interactions. The biomolecular corona is dynamic and has various implications on the in vivo fate of NPs, affecting their circulation time,^[^ ^1^ ^]^ cellular interactions,^[^ ^2^ ^]^ biodistribution^[^ ^3^ ^]^ and targeting ability,^[^ ^4^ ^]^ amongst others. Depending on the physicochemical properties of the NPs, the resulting biomolecular corona can induce aggregation, encourage opsonin recruitment as well as bring about conformational changes in the constituent proteins.^[^ ^5^ ^]^ This can lead to phagocytosis, inflammatory responses, and eventually immune system‐based clearance of NPs.^[^ ^6^ ^]^ Strategies toward controlling protein corona interactions are therefore highly desired.

Polyethylene glycol (PEG) is a well‐known surface passivation agent that limits the extent of protein adsorption. Cui *et al.*, elaborated the advantage of using PEG as the basis of NP system instead of PEGylation,^[^ ^7^ ^]^ where NPs assembled from PEG, circumvent the spatial challenges of PEGylation as well as reduce the extent of the corona formation, thereby emerging as an optimal system to explore nano‐bio interface interactions.^[^ ^8^, ^9^ ^]^ Whilst the low‐fouling characteristics of PEG reduces the corona, for minimization of immune responses, it does not prevent it in its entirety.^[^ ^10^, ^11^ ^]^ The protein corona is not necessarily bad, where the enrichment of clusterin on both PEGylated and non‐PEGylated NPs contributes to their stealth properties.^[^ ^10^, ^12^ ^]^ Complement factor C3 in the corona of dextran‐coated NPs enables inherent B‐cell targeting without the need for active targeting moieties.^[^ ^13^ ^]^ Polycatecholamine coated NPs, on the other hand, can bind coagulation proteins like fibrinogen, triggering coagulation cascades and accelerated clearance.^[^ ^14^ ^]^ This emphasizes that enrichment of certain proteins in the corona has functional consequences. This approach, which can be termed as functional biomolecular coronas, can enhance NP performance by modulating interactions with biological systems. Taking it one step ahead, deliberate direct or indirect enrichment with a protein can bring about desirable functional consequences. As shown previously, pre‐coating NPs with selected proteins prior to their exposure to the biological environment enables directing the formation of a protein corona that can enable cell targeting, stealth or specific uptake.^[^ ^15^ ^]^ One of the main challenges of the pre‐coating approach, however, is that the protein should be retained and accessible in the outer corona layer for further downstream NP‐cell interactions.^[^ ^16^ ^]^ This can be accomplished by modifying the surface with an “anchor”. For instance, the surface modification of liposomes with amyloid β‐derived peptides (an anchor), recruited plasma apolipoproteins on to the NP surface and enabled targeted delivery to brain tissue.^[^ ^17^ ^]^ This proof‐of‐concept demonstrates the potential to engineer NP surfaces by strategically incorporating specific proteins into the corona. We hypothesize that combining pre‐coating and anchoring strategies can provide greater control over the composition and function of the protein corona.

Along with the aforementioned proteins, coagulation proteins are a prominent constituent of the biomolecular corona.^[^ ^5^ ^]^ The interaction of NPs with the coagulation system remains a crucial, yet understudied aspect of their safety profile,^[^ ^18^ ^]^ where NPs can trigger unwanted coagulation‐mediated effects depending on the physicochemical properties of the NPs.^[^ ^19^ ^]^ Adsorption or binding of coagulation proteins on the NP surface can initiate contact activation of the coagulation factors, leading to thrombus formation on the surface.^[^ ^18^, ^20^ ^]^ While abundant coagulation proteins within the corona can lead to opsonization and rapid immune clearance, a specific protein like antithrombin III (ATIII), which is also a prominent component of the biomolecular corona, can offer potential benefits as it is a well‐established anticoagulant acting along the coagulation cascade. ATIII exhibits additional anti‐inflammatory properties through its interactions with leukocyte surface receptors.^[^ ^21^ ^]^ This enables mitigation of inflammatory and coagulation subsets of the immune system.

Herein, we leverage heparin, a natural binding partner of ATIII, as an anchor strategy for the stable and deliberate enrichment of ATIII in the corona of the NPs.^[^ ^22^, ^23^, ^24^ ^]^ We develop synthetic PEG NPs with conjugated heparin, such that the heparin retains its in‐blood activity, and focus on modulating corona composition through the binding of a functional anticoagulant biomolecule. Although a PEG‐based NP system has an inherent low‐fouling character that reduces the overall corona, it cannot circumvent the potential for coagulation effects mediated by the relevant biomolecules. Thereby with our NP system, our goal is to leverage ligand‐receptor (Heparin‐ATIII) binding to enrich the protein corona with components (ATIII) that can mitigate coagulation responses induced by PEG, whilst retaining stealth. The work is arranged by 1) synthesis of the hybrid NPs, 2) investigation of targeted ATIII binding, 3) analysis of changes to the extent and composition of the protein corona, and 4) resulting hemocompatibility of the NPs.

Through targeted binding of ATIII to the PEG NPs via the heparin complex, we confer anticoagulant properties to the NPs, whilst retaining the inherent stealth of PEG. By focusing on specific protein recruitment within the corona, we can achieve a more comprehensive approach for designing NPs that are both stealthy and exhibit desirable anticoagulant properties. Our strategy therefore has high utility toward cloaking synthetic nanoparticles with invisibility toward the immune system in human blood, potentially leading toward greater efficacy in nanomedicine.

## 2 Results and Discussion

### 2.1 Synthesis and Characterization of MS‐ and PEG‐ NPs

**Figure 1:**
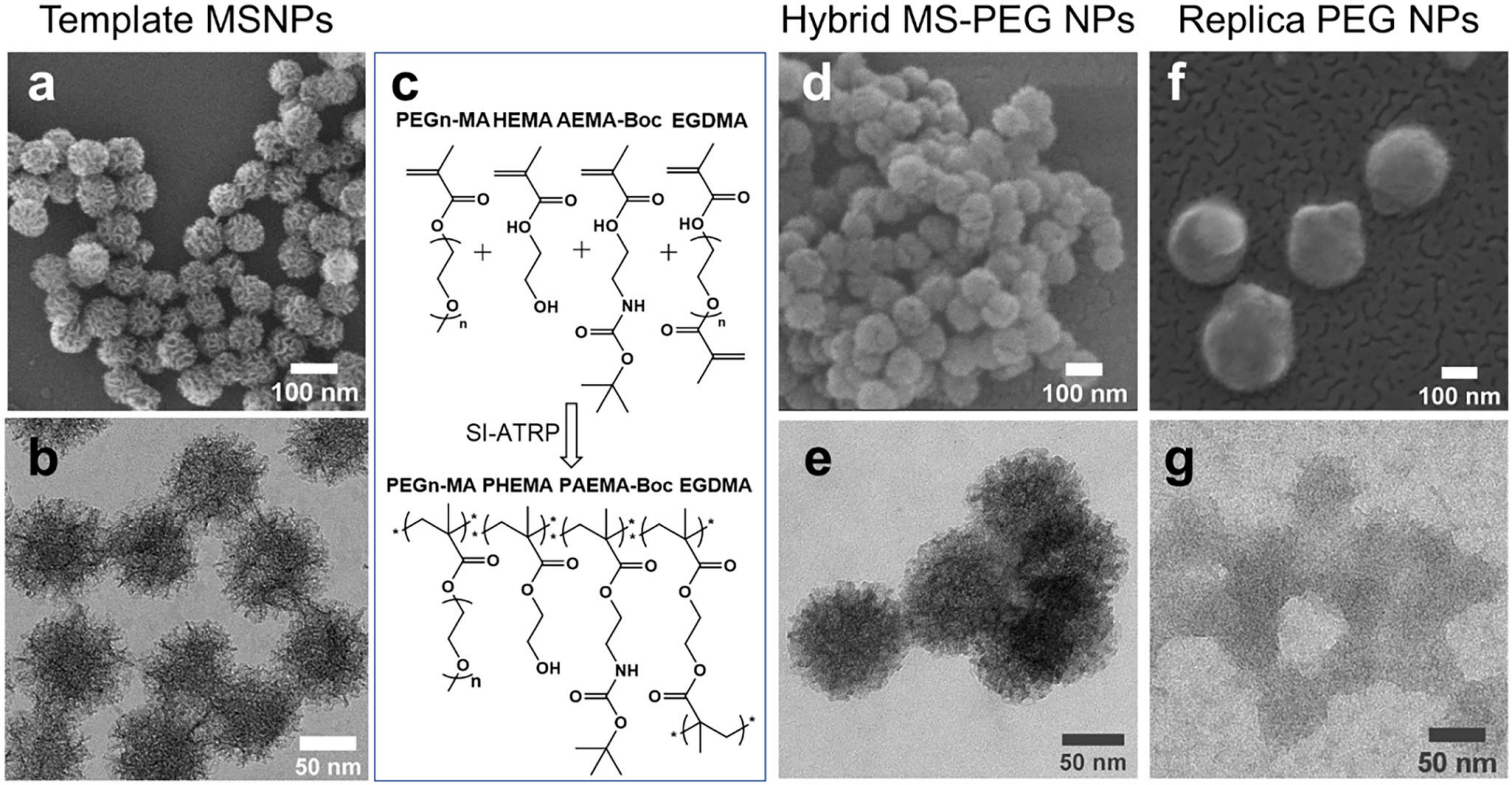
a. SEM and b. TEM characterization of the template MSNPs c. Schematic illustration of PEG replica NPs synthesis by SI‐ATRP, with functional monomers and crosslinker. d,f. SEM and e,g. TEM characterisation of hybrid MS‐PEG NPs and replica PEG NPs.

PEG, being the gold standard low‐fouling polymer, was chosen as backbone for synthesizing highly crosslinked NPs with an inherent low‐fouling character. For the fabrication of PEG‐NPs, mesoporous silica NPs (MSNPs) were used as sacrificial templates, according to a modified literature method.^[^ ^25^, ^26^ ^]^ The MSNPs were ≈90 nm in diameter and had a zeta potential of ≈−18 mV. The porous structure and spherical morphology of the MSNPs were observed with electron microscopy (**Figure** **1**a,b). Following synthesis and purification, the MSNPs were aminated with 3‐Aminopropyl‐triethoxysilane (APTES) and subsequently modified with the atom transfer radical polymerization (ATRP) initiator α‐bromoisobutyryl bromide. The hybrid particles were obtained by infiltration of chosen monomers in the pores of the MSNPs, followed by polymerization by surface‐initiated atom transfer radical polymerization (SI‐ATRP). The PEG‐based monomer, poly (ethylene glycol) methyl ether methacrylate (PEG‐MA) formed the major constituent of the NPs. 2‐hydroxyethyl methacrylate (HEMA) and 2‐Boc‐aminoethyl methacrylate(AEMA‐Boc) were included to incorporate hydroxyl and amine functional handles respectively. Ethylene glycol dimethacrylate (EGDMA), acted as crosslinker, providing the required structural integrity that holds the NP network together after dissolution of the silica. We note that the polymerization occurs all over the MSNPs, not only inside the pores. Absence of the sharply defined pores in the MSNPs as in Figure 1b due to polymerization, observed in scanning electron microscopy (SEM) and Transmission Electron Microscopy (TEM) (Figure 1d,e) confirmed successful polymerization. Subsequently, **controlled hydrofluoric (HF) acid etching of the MSNP template yielded the crosslinked replica PEG‐NPs**. SEM revealed monodisperse, spherical particles of ≈110 nm in diameter (Figure 1f) while DLS analysis revealed an increase in hydrodynamic particle size in the solvated state (Table S1, Supporting Information), likely due to the long surface‐tethered chains creating a greater drag on the particles in solution. This swelling behavior characteristic of polymeric NPs indicates their soft structure and polymeric composition.^[^ ^27^, ^28^ ^]^

The PEG replica NPs had a zeta potential of −0.1 mV indicating shift from a negatively charged silica toward a more neutral charge. The dissolution of the template was also confirmed by the lack of contrast and the absence of structured pores characteristic of the sacrificial silica template in the TEM images (Figure 1g; Figure S1, Supporting Information). The dissolution of the rigid template MSNP was further verified by height profile measurements using Atomic Force Microscopy (AFM) operating under peak force tapping method (Figure S2, Supporting Information). A reduced height of 14.8 ± 6.4 nm was measured for PEG NPs compared to the 79.4 ± 2.7 nm of the initial MSNP (Figure S2, Supporting Information), indicating a soft structure of the PEG NPs.

The inclusion of HEMA and AEMA‐Boc were confirmed by successful subsequent functionalization with N‐hydroxysuccinimide (NHS)‐activated Alexa Fluor 488 (AF488‐NHS) via the hydroxyl of HEMA and AEMA was used for modification with heparin. The NP counts, as estimated by Nanoparticle Tracking Analysis, were typically in the range of 5.2 ×10^10^–1.5 × 10^11^ particles/mL.

Among the heparins, low molecular weight heparins (LMWHs) have been reported to have less binding to plasma proteins while maintaining a favorable pharmacological profile.^[^ ^29^ ^]^ Tinzaparin sodium (mol. wt. 6500 Da), the largest of the LMWHs, was chosen to retain the balance between antithrombotic activity and desired low fouling character. Tinzaparin, was covalently attached to the AEMA functional handles via amide bond formation, mediated via N, N'‐dimethylmethylenediamine (DMTMM). As indicated in **Table** **1**, the AEMA deprotection step led to a positive shift in the zeta potential of the NPs due to the presence of the exposed primary amine groups. The DMTMM‐activated heparin was then conjugated to the amine groups. Similar to unfractionated heparin, tinzaparin possesses a negative charge. Consequently, the incorporation of tinzaparin (PEG‐HEP) shifted the overall zeta potential of the NPs toward negative as compared to the unmodified PEG‐NPs. The zeta potential measurements for each modification step are summarized in Table 1. To confirm heparin conjugation to PEG NPs, fluorescent labeling of both components was performed followed by confocal laser scanning microscopy (CLSM) to determine colocalization. A Pearson's correlation coefficient of r = 0.712 revealed a significant positive relationship between the fluorescence signals, confirming the successful conjugation of heparin to the PEG NPs (Figure S3, Supporting Information). Quantitative fluorescence analysis revealed a heparin content of ≈43 ± 4 pg/NP, which is in alignment with the quantity of AEMA expected per NP (Figure S4a, Supporting Information). Furthermore, Fourier transform infrared spectroscopy (FTIR) analysis confirmed the presence of heparin on the NPs, and the emergence of a new amide peak corresponding to the coupling between heparin (carboxylic acid) and the AEMA (amine) monomer (Figure S4b, Supporting Information).

**Table 1:** Zeta potentials of the different NP systems.

NP system	Zeta potential [mV]
MSNP	−18
MSNP‐αBiBB	−4.6
PEG	−0.1
PEG (AEMA deprotected)	+12
PEG‐HEP	−22

### 2.2 Heparin Incorporation Imparts Anticoagulant Character to NPs in Vitro

**Figure 2:**
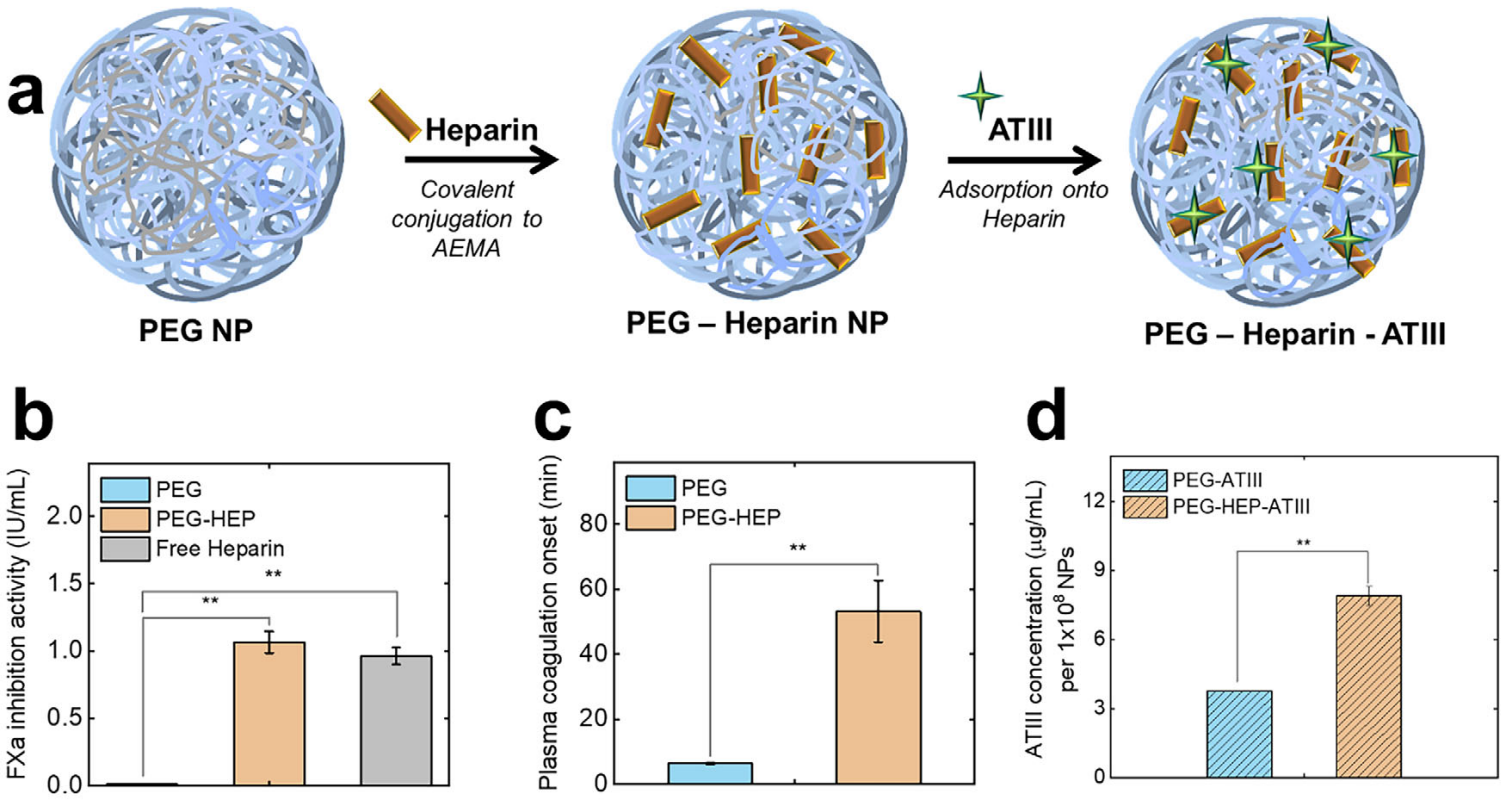
a. Heparin conjugation and ATIII adsorption reaction scheme. b. Calculated activity of conjugated heparin on NP (PEG‐HEP) determined by inhibition of coagulation factor FXa against 1U mL^−1^ anti‐FXa‐activity free heparin in citrated plasma, measured in a chromogenic assay. c. Coagulation time in minutes of recalcified citrate plasma at 37 °C in the presence of PEG and PEG‐HEP NPs. d. Concentration of bound ATIII on NP (PEG‐HEP‐ATIII) determined by its activity of inhibition of coagulation factor FXa in PBS buffer system measured in a chromogenic assay. Data is represented as a mean ± standard error of *n* = 3. Statistical significance is determined by one‐way ANOVA followed by Tukey's test and is represented as **p < 0.001.

A functional bioassay measuring the Factor Xa (FXa) inhibition activity confirmed the presence and bioactivity of the conjugated heparin (**Figure** **2**b; Figure S5 and S6, Supporting Information). This involved a chromogenic evaluation of PEG‐HEP NPs for anticoagulant performance in human plasma. Unmodified PEG NPs did not inhibit FXa. Conjugation of heparin to PEG NPs did not affect the inhibitory capacity of the heparin, where the 1 × 10^8^ PEG‐HEP NPs exhibited an activity corresponding to 1 U mL^−1^ activity of free heparin. Given that the conjugation strategy was performed in an excess of heparin, an effect of heparin conjugation density on the inhibitory capabilities of our NP system is not anticipated, where a grafting‐to method is not anticipated to produce densely conjugated polymer brushes. Whilst our strategy for conjugating heparin has maintained its activity, this will not necessarily hold for other conjugation strategies.

Additionally, the anticoagulant efficacy of heparin bound to NPs was investigated by measuring the increase in clotting time of plasma upon exposure to the NPs. The PEG‐HEP NPs, being anticoagulant, had a delayed clot onset time of 53 min, while the unmodified PEG NPs exhibited a clotting onset time of ≈7 min (Figure 2c). Coagulation time of plasma without NPs was 11 ± 3 min while citrate plasma supplemented with 1 U mL^−1^ heparin did not clot over the recorded time period of the experiment.

### 2.3 ATIII Incorporation Induces Minimal Changes to the Protein Corona Composition

ATIII works synergistically with heparin to inhibit thrombin. This natural binding enabled the incorporation of ATIII onto the heparin‐modified NPs, which is achieved through an assembly process (not covalently attached). The anti‐FXa assay was used to quantify the bound ATIII and ascertain its functionality. The bound ATIII on PEG‐HEP was estimated to be 7.9 µg mL^−1^ per 1 × 10^8^ NPs while for ATIII adsorbed on PEG was 3.8 µg mL^−1^ per 1 × 10^8^ NPs (Figure 2d). Following the successful adsorption and quantification of ATIII onto the PEG and PEG‐HEP NPs, these functionalized nanoparticles were subsequently exposed to human plasma for investigations into their biomolecular corona.

Upon incubation in plasma, the size distribution of PEG‐ATIII and PEG‐HEP‐ATIII nanoparticles widened with a characteristic broadened main peak and the appearance of a minor population of larger aggregates, indicative of protein corona formation (Figure S7, Supporting Information). A comprehensive and quantitative characterization of the biomolecular corona was subsequently performed using sodium dodecyl sulfate polyacrylamide gel electrophoresis (SDS‐PAGE) analysis, bicinchoninic acid (BCA) assay, and quantitative proteomics. Figure S8a (Supporting Information) shows the SDS PAGE gels obtained from proteins present in the hard corona of the PEG, PEG‐ATIII, PEG‐HEP, and PEG‐HEP‐ATIII. The minimal band pattern (Figure S8a, Supporting Information) across all systems confirms the low‐fouling nature, despite the presence of heparin and ATIII. The BCA assay (Figure S8b, Supporting Information) further corroborates this, with negligible protein adsorption onto the NPs compared to the high protein concentration (60–80 mg mL^−1^) of the incubation medium (plasma). Furthermore, the “hard” corona composition of three replicates of each system was analyzed by mass spectrometry, following 1 h incubation in human plasma. As described previously, the biomolecular corona was evaluated based on a number of identified proteins as well as the relative abundance of the enriched proteins.^[^ ^25^ ^]^ The number of identified proteins remained similar (**Table** **2**), with PEG and PEG‐HEP having the lowest number of total identified proteins in the corona. The decoration of the PEG NPs with ATIII marginally increased the number of proteins in the corona, though this reduced for the PEG‐HEP‐ATIII NPs. Label‐free quantification (LFQ) intensity data (Table 2) confirmed the successful incorporation of ATIII onto both PEG and PEG‐HEP NPs. Given the abundance of ATIII in plasma, it was detected in the corona of all nanoparticle types. However, PEG‐HEP NPs exhibited higher ATIII LFQ intensity than PEG NPs, consistent with the natural affinity between ATIII and heparin. This natural recruitment also has likely contributed to the lower F1 + 2 levels of PEG‐HEP NPs. Notably, the deliberately ATIII‐decorated NPs exhibited significantly higher (≈6‐fold higher) relative intensity levels of ATIII compared to their respective PEG‐HEP and PEG counterparts, demonstrating that after decorating the NPs with ATIII, the ATIII was able to remain in the hard corona after immersion into human plasma.

**Table 2:** Total number of identified proteins in the biomolecular corona of each NP system after incubation of the NPs in human plasma for 1 h and observed relative abundance (label free quantification intensity) of ATIII in protein corona of NPs.

NP system	Total number of identified proteins	Log_2_ (LFQ Intensity of ATIII)
PEG	606 ± 49	20.39 ± 0.42
PEG‐ATIII	647 ± 26	26.43 ± 0.17
PEG‐HEP	639 ± 24	21.25 ± 0.01
PEG‐HEP‐ATIII	588 ± 66	27.92 ± 0.93

**Figure 3:**
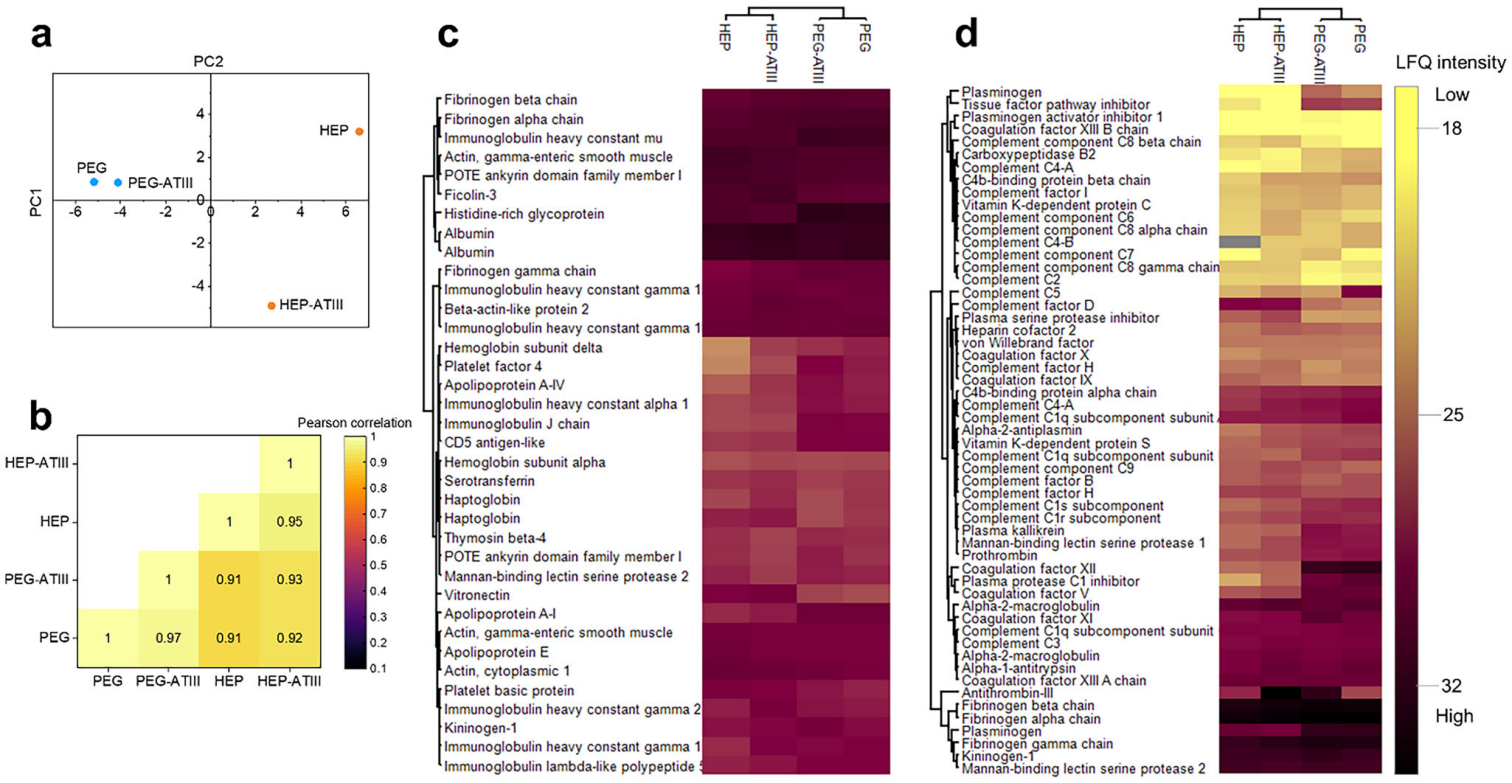
Analysis of the mass spectrometry data via a. PCA of the first two components covering 74% of the data b. Pearson correlation of proteins identified in the protein corona of NP systems. c. Enrichment analysis of top 20 abundant proteins in the protein corona of at least 70% across the NP systems d. Enrichment analysis of the abundant complement and coagulation associated proteins in the protein corona. Data are presented as averages across all replicates. For enrichment plots, lighter colours represent low intensity.

Further investigations into the composition were performed via principal component analysis (PCA), Pearson correlation, and enrichment analysis. PCA analysis (**Figure** **3**a) of the first two principle components revealed differences between the PEG and PEG‐HEP coronas, where 74% of the variability could be covered by the first two components. The corona of PEG‐ATIII is similar to the corona of PEG, while the PEG‐HEP‐ATIII corona differs from the PEG‐HEP across one principle component. Therefore, HEP‐ATIII decoration seems to bring about differences in the protein enrichment degree within the corona. Pearson correlation (Figure 3b) revealed 90% similarity in the overall biomolecular corona composition between PEG and PEG‐HEP NPs. Despite the decoration of PEG with heparin as well as ATIII, the coronas of the NP systems remain similar, highlighting the inherent low‐fouling character of PEG. The 10% dissimilarity, corresponding to 38 proteins with significantly different intensities has been visualized in the volcano plot (Figure S9 and Table S2, Supporting Information). Note that the differences stem from variations in relative abundance and not absence or presence of the proteins. PEG‐HEP NPs exhibited a relative reduction in the abundance of proteins associated with potential bio‐adverse effects, such as coagulation factors (e.g., Coagulation factor XII, Prothrombin) and immunoglobulins. This altered protein adsorption profile on PEG‐HEP NPs may contribute to their reported in‐blood behavior.

All NP types displayed a high degree of similarity in the corona. Aligning with literature, albumin, immunoglobulin, apolipoproteins, and fibrinogen were observed as the abundant proteins across all NP systems (Figure 3c).^[^ ^30^ ^]^ This consistency suggests that the biological effects brought about by these proteins will likely be similar across different NP systems. While the enrichment of these proteins and their individual effects on hemocompatibility and cell uptake have been studied, their collective impact within a complex protein corona is less straightforward.^[^ ^31^ ^]^ Dysopsonins like albumin and apolipoproteins can hinder cellular uptake, while immunoglobulins and complement factors can promote it.^[^ ^31^, ^32^, ^33^ ^]^ Immunoglobulin enrichment often triggers C3 deposition and complement activation. The combined effects of multiple proteins within the corona can be complex, intricate, and synergistic. The NPs in this study maintain an overall favorable hemocompatibility and immune cell association profiles.

Among all these commonly found proteins in the hard corona, our system also made ATIII one of the abundant proteins. Being one of the abundant proteins in plasma, ATIII was also present in corona of the unmodified PEG‐NPs.^[^ ^34^, ^35^, ^36^ ^]^ The presence of ATIII in the corona of unmodified PEG NPs ascertains its prevalence in the biological milieu encountered by the NPs. Enrichment analysis of coagulation and complement proteins (Figure 3d) revealed minor variations in corona proteins, as observed between PEG‐based (PEG and PEG‐ATIII) and PEG‐HEP (PEG‐HEP and PEG‐HEP‐ATIII) NPs. These included Coagulation Factor XII, Histidine rich glycoprotein, and TFPI. Notably, these proteins were present in heparin inclusive NP systems and were not enriched in the biomolecular corona around the ATIII‐decorated particles. Therefore, their presence likely contributes more to the affinity to the underlying heparin (PEG‐HEP) rather than the specific effect induced by ATIII decoration. Additionally, PEG NPs displayed higher enrichment of complement factor C5 which is an abundant protein in plasma. While both ATIII‐modified NPs (PEG‐ATIII and PEG‐HEP‐ATIII) displayed significantly higher levels of ATIII compared to their unmodified counterparts (PEG and PEG‐HEP), the overall protein composition of the corona remained similar. The slight differences in the intensities of the identified proteins might have contributed to the difference observed in the PCA of the PEG‐HEP and PEG‐HEP‐ATIII. According to the enrichment analysis, ATIII was the only major compositional difference identified between the coronas of these NP systems. Despite this apparent similarity, functional assays revealed differences in the biological profiles of the NPs upon ATIII decoration.

### 2.4 ATIII Influences the Immune Response and Confers ex Vivo Anticoagulant Character

Following the characterization of the corona, the functional impacts of the corona were explored in ex vivo whole blood assays for immune cell association and hemocompatibility. Human blood assays recapitulate a biologically relevant environment that enables concurrent examination of particle‐cell interactions in the context of the biomolecular corona formation.

**Figure 4:**
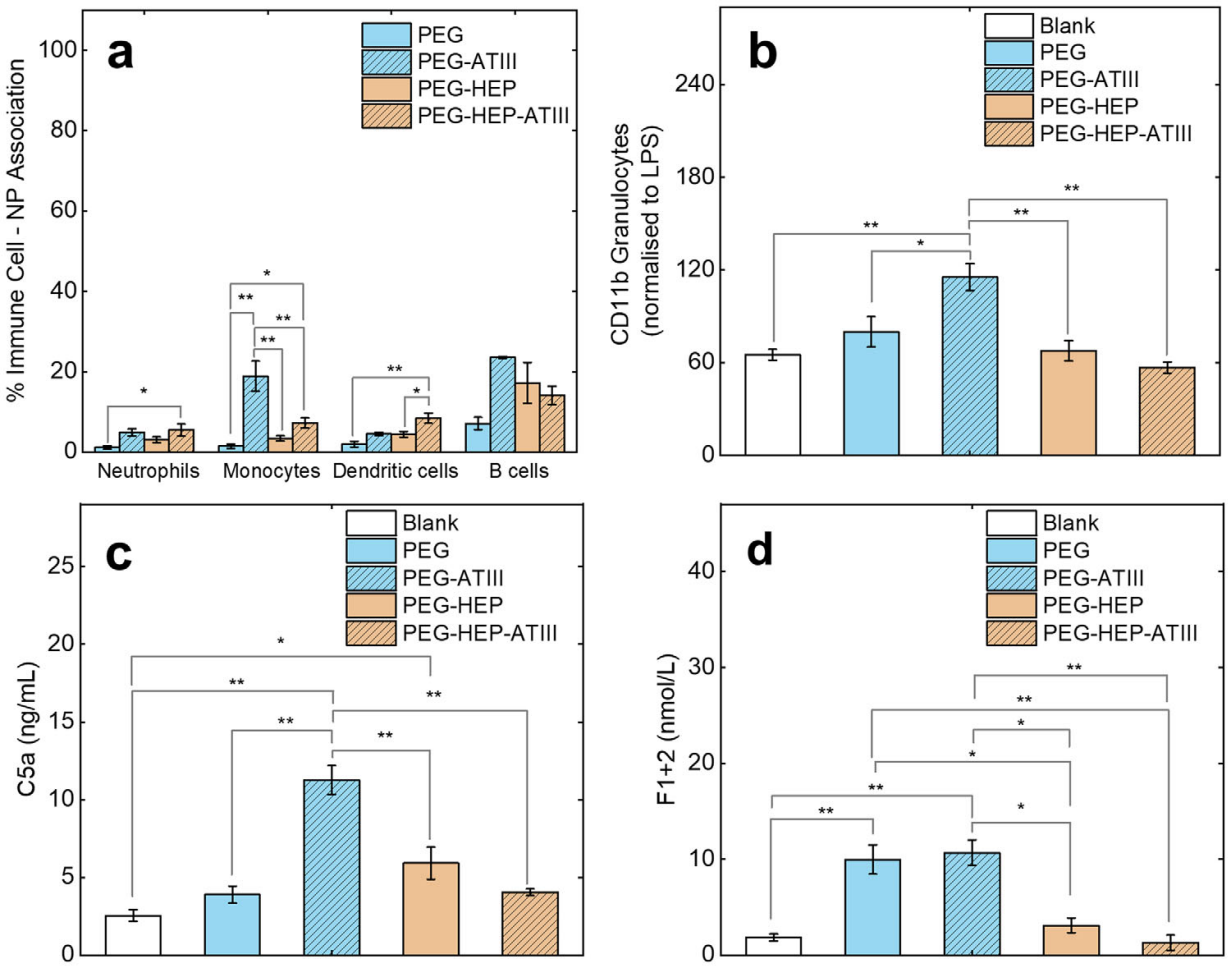
a. Cellular association of ATIII‐decorated PEG and PEG‐HEP NPs with neutrophils, monocytes, dendritic cells, and B cells in whole blood. b–d. Inflammation and coagulation activation of blood in response to NPs incubated for 2h under quasi‐static conditions via – b. CD11b expression on granulocytes as a marker of leukocyte inflammation (normalized to 100 units mL^−1^ lipopolysaccharide which serves as the positive control) c. complement fragment C5a as a marker of humoral inflammation and d. Prothrombin fragment F1 + 2 as a marker of plasmatic coagulation. Data are represented as mean ± standard error; *n* = 3. Statistical significance is determined by one‐way ANOVA with Tukey's comparison test and is represented as *p < 0.05, **p < 0.001.

Immune cell associations were investigated for PEG, PEG‐HEP, PEG‐HEP‐ATIII NPs following incubation in human whole blood, as reported previously (**Figure** **4**a).^[^ ^25^ ^]^ Each particle system was fluorescently labeled through AF488‐NHS with the HEMA functional handle included in the polymer matrix. After 1 h incubation at 37 °C, cell association was determined by flow cytometry through individual phenotyping of the cells, as published previously (Figure S10, Supporting Information).^[^ ^25^ ^]^ As expected, PEG NPs retained a stealthy character, showing an association below 5% for neutrophils, monocytes, and dendritic cells (T‐cells and NK cells in Figure S11, Supporting Information). NP and B cell association was slightly higher ranging from 7 to 20%. A similar trend was observed by Weiss, et al., and was shown to be originating from complement proteins bound to the nanoparticle surface that mediate interactions with B cells.^[^ ^8^, ^25^ ^]^ The association trend observed with PEG‐HEP was similar to the PEG‐NPs, suggesting a consistent stealth endowed by the low‐fouling PEG, independent of the heparin moiety. PEG‐HEP‐ATIII NPs had slightly higher associations than the PEG and PEG‐HEP counterparts. Confocal laser scanning microscopy (CLSM) (Figure S12, Supporting Information) showed that the percent of PEG‐HEP‐ATIII NPs that were associated with leukocytes, were mostly internalized by the associating cell. This association could be attributed to potential immunomodulation through the biomolecular corona.^[^ ^21^, ^37^, ^38^ ^]^ The PEG‐ATIII NPs exhibited significantly higher colocalization with monocytes. We hypothesize that presence of ATIII on the NPs can enable their interactions with leukocyte receptors, perhaps through binding to cell surface glycosaminoglycans.^[^ ^38^, ^39^ ^]^ Alternatively, constituents of the biomolecular corona formed on the ATIII NPs can instigate passive targeting of PEG‐ATIII to leukocyte subsets.

The nanoparticle set was evaluated for hemocompatibility in whole human blood following established protocols.^[^ ^19^ ^]^ After 2 h incubation, blood was assessed for hemostasis and inflammation markers. As elaborated in Weber et al., biomaterial‐blood interactions can induce cellular and humoral reactions, leading to inflammation, complement, and coagulation activation.^[^ ^40^ ^]^ Accordingly, our evaluation includes a comprehensive hemocompatibility assessment including cellular responses, monitored via markers such as CD11b and CD62P, as well as humoral factors, specifically coagulation activation (F1 + 2), complement activation (C5a), and platelet activation (PF4).

CD11b expression on neutrophils was used as a leukocyte activation marker of inflammation. PEG‐ATIII NPs induced the highest granulocyte activation, followed by PEG NPs while PEG‐HEP displayed lower activation compared to PEG NPs (Figure 4b). PEG‐HEP‐ATIII significantly reduced neutrophil activation, with levels comparable to blank (blood without NPs), highlighting a potential of the heparin‐ATIII conjugate to mitigate cellular inflammatory responses.

Monocyte activation, as measured by CD11b levels, was the highest for PEG‐ATIII while PEG‐HEP‐ATIII displayed a trend toward lower activation (Figure S13a, Supporting Information). Platelet activation was not observed in response to the NPs. PEG‐HEP‐ATIII NPs exhibited the highest blood platelet activation, as measured by CD62P levels (Figure S13b, Supporting Information). However, this activation did not lead toward release of platelet factor 4 (PF4) (Figure S13c, Supporting Information).

The C5a fragment of the complement pathway was used as a humoral inflammation marker (Figure 4c). PEG NPs, did not induce a significant increase in C5a levels, despite a higher C5 deposition in its corona. NPs decorated solely with ATIII (PEG‐ATIII) exhibited a pro‐inflammatory effect, as evidenced by elevated C5a levels. PEG‐HEP NPs induced significantly higher C5a levels compared to blank. Notably, ATIII incorporation in presence of heparin (PEG‐HEP‐ATIII) effectively reduced complement activation, as compared to PEG‐ATIII.

Corroborating the plasma recalcification test, PEG NPs induced relatively higher prothrombin fragment F1 + 2 levels, indicating a coagulant nature (Figure 4d), meaning PEG can activate coagulation cascade. The observed coagulant behavior of PEG emphasizes a critical point that stealth may not always mean hemocompatibility. While traditional nanoparticle design often prioritizes creating low‐fouling NPs for reduced cellular activation effects, our findings highlight the importance of evaluating potential interactions with the coagulation cascade. The incorporation of heparin, being an anticoagulant molecule, lowered the F1 + 2 levels. PEG‐HEP‐ATIII displayed the lowest F1 + 2 generation, comparable to blank (Figure 3d).

These findings suggest that the combination of heparin and ATIII effectively counteracts the coagulant effect observed with PEG NPs. The addition of ATIII alone (PEG‐ATIII) did not achieve this effect. The presence and relative abundance of heparin‐ATIII within the corona likely drives the observed biological effects, significantly enhancing hemocompatibility while simultaneously retaining PEG's desirable stealth properties. These observations highlight the critical role of the synergistic interaction between heparin and ATIII in achieving a desirable anticoagulant effect.^[^ ^41^ ^]^ Figure 3d therefore demonstrates a four‐fold improvement in the hemocompatibility, in terms of coagulation effects, of the PEG‐HEP‐ATIII NPs. Crucially, this benefit was achieved without inducing adverse thromboinflammatory reactions, as evidenced by no significant changes in CD11b expression or C5a levels. This is a significant enhancement of hemocompatibility, resulting from an engineered protein corona.

Surprisingly, the overall protein corona composition remained largely unaffected by the heparin‐ATIII complex, with a high degree of similarity (90% Pearson correlation) between PEG and PEG‐HEP‐ATIII NPs. This indicates that the complex can be stably incorporated without disrupting the endogenous corona, suggesting the underlying PEG largely governs the overall corona formation. The variations observed in PCA analysis can be attributed to slight differences in LFQ intensities While identifying specific proteins within the corona provides valuable insights, a direct causal link between corona composition and functional improvements cannot always be definitively established. This underscores the necessity of assessing the biological consequences of the adsorbed proteins. Our study, therefore, demonstrates the potential to functionally leverage the protein corona by strategically anchoring and enriching specific proteins, thereby conferring desired properties. This targeted engineering approach can improve the hemocompatibility of the NPs without compromising their stealth properties.

## 3 Conclusion

This study highlights the importance of designing NPs with an inherent low‐fouling and stealthy character, as well as accounting for hemocompatibility. By using a PEG‐based system, we could achieve low‐fouling properties while still maintaining space for attaching functional molecules to the NPs. Our findings demonstrate that PEG's ability to promote a stealthy effect and reduce cellular activation is preserved even after decorating the NPs with biomolecules. However, the research goes beyond simply achieving stealth.

We highlight the critical need to engineer a functional biomolecular corona, specifically focusing on immunomodulation via coagulation mitigation. The influence of NPs on coagulation is often overlooked, unlike with traditional blood‐contacting biomaterials, yet it can potentially offset the intended benefits of a stealthy design. Our work suggests that solely minimizing the corona through PEG, while beneficial for reducing immune responses, is insufficient for fully mitigating interactions with coagulation cascade proteins. Therefore, we propose engineering an anticoagulant corona to prevent adverse coagulation reactions at the nano‐bio interface, serving as a prime example of a functional corona. Decorating NPs with heparin, which itself is an anticoagulant molecule, offers a platform for the controlled recruitment of additional coagulation proteins. However, heparin's broad binding profile can dampen its anticoagulant effect within the corona. To address this limitation, we introduce a more targeted approach: deliberately pre‐enriching the corona with ATIII, a key regulator of coagulation, via its specific interaction with heparin.

Leveraging the natural high‐affinity interaction between heparin and ATIII resulted in a biomolecular corona specifically enriched with a protein complex capable of actively countering coagulation reactions at the nano‐bio interface. Our ATIII‐functionalized hybrid NPs successfully reduced the coagulation effects that were instigated by the naked PEG NPs. Our approach, encompassing both immunomodulation and hemocompatibility within the context of biomolecular corona engineering, reveals an extra dimension of interactions of nanomaterials with human blood that should be considered for designing therapeutic NPs. This work can potentially inform safer NP designs, more efficacious therapeutic applications, and, consequently, improved therapeutic efficiency.

## 4 Experimental Section

### Materials

Analytical grade chemicals were used as received without further purification except for monomers. All liquid monomers were purified through an aluminum oxide column prior to polymerisation to remove the inhibitor. High purity water (Milli‐Q water) with a resistivity greater than 18.2 MΩ cm was obtained from a three‐stage Millipore Milli‐Q Plus 185 purification system (Millipore Corporation, USA). Synthesis of MSNPs was carried out using cetyltrimethylammonium bromide (CTAB), ammonium hydroxide solution (NH_3_, 28−30%), and tetraethyl orthosilicate (TEOS, 98%) obtained from Sigma Aldrich. Silica particle modification was carried out using (3‐aminopropyl)‐triethoxysilane APTES, 98%), pyridine (anhydrous, 99.8%), tetrahydrofuran (THF, anhydrous, 99.9%), and α‐bromoisobutyryl bromide (98%), which were all purchased from Sigma Aldrich. For the SI‐ATRP, poly (ethylene glycol) methyl ether methacrylate (PEGMA), ethylene glycol dimethacrylate (EGDMA, 95%), (2‐Boc‐amino) ethyl methacrylate (AEMA‐Boc), 2‐hydroxyethyl methacrylate (HEMA, 97%), N, N, N′, N″, N″‐pentamethyldiethylenetriamine (PMDETA, 99%), CuBr (98%), and nitric acid (70%) from Sigma Aldrich were used. Template removal was performed using hydrofluoric acid (HF, 48%) and ammonium fluoride (NH_4_F, 98%) from Aldrich. Fluorescence labeling was carried out using AF488‐NHS, purchased from Thermo Fisher, and dimethyl sulfoxide (DMSO, anhydrous, >99%) obtained from Aldrich. The heparin utilized was innohep multi 10 000 anti‐FXa I.E./mL (Leo Pharma, Denmark). Human Antithrombin III was purchased from CellSystems GmbH (Germany). To measure heparin activity, COAMATIC Heparin test kit was purchased from Chromogenix, Italy.

For cell phenotyping, antibodies against CD3 AF700 (SP34‐2), CD11b Pacific Blue (ICRF44), CD14 APC‐H7 (MΦP9), CD66b BV421 (G10F5), CD45 V500 (HI30), CD56 PE (B159), lineage‐1 cocktail APC, HLADR PerCP‐Cy5.5 (G46‐6), and CD19 BV650 (HIB19) were used, all purchased from BD Biosciences, except for lineage‐1 cocktail and CD19, which were obtained from BioLegend (CA, USA). For hemocompatibility testing, standard heparin sodium was purchased from Ratiopharm, Germany, and C‐reactive protein test kit (point of care test) from MöLab, Germany. The flow cytometry antibodies PE/Cy7, CD11b‐PacificBlue (clone ICRF44) were purchased from Biozol; CD14‐APC (clone M5E2), CD62P‐PE (clone AK4) and CD41a‐FITC (clone HIP8) were obtained from BD Biosciences, USA. C5a, prothrombin fragment F1 + 2, and platelet factor 4 (PF4) concentrations were determined using ELISA kits from different distributors: C5a ELISA from DRG Diagnostics, Marburg, Germany; Enzygnost F1 + 2 from Siemens Healthineers, Erlangen, Germany; Zymutest PF4 kit from CoaChrom Diagnostica GmbH, Austria. Biomolecular corona characterization was carried out using DPBS, ammonium bicarbonate (NH_4_HCO_3_, 99%), DL‐dithiothreitol (99%), iodoacetamide, trifluoroacetic acid (99%), purchased from Sigma Aldrich and sequencing‐grade modified trypsin purchased from Promega (Madison, WI). Formic acid (95%) and acetonitrile (CH_3_CN, 99.5%) were purchased from Merck.

### PEG‐NP Synthesis, Characterization and AF488 Conjugation

PEG replica NPs were fabricated via template‐mediated SI‐ATRP, according to a modified literature method.^[^ ^27^ ^]^ In brief, MSNPs synthesized based on a previously reported method^[^ ^7^ ^]^ served as the templates. The templates were then modified with α‐bromoisobutyryl bromide, an ATRP initiator, and subsequently infiltrated with PEG‐based monomers. A molar ratio of MSNP:monomers:CuBR:PMDETA of 2:1000:2:2 and a monomer ratio PEGMA:HEMA:AEMA‐Boc:EGDMA of 84:3:3:10 was used for the polymerization reaction. The reaction mixture was degassed via 4 freeze‐pump‐thaw cycles, and following PMDETA injection, polymerization was conducted at 50 °C for 20 h in an oxygen exclusive environment. The reaction was quenched by exposing to air and cooling down to room temperature. Purification was carried out by repeated centrifugation (10 000 g, 5 min) and resuspension steps in nitric acid (1 M, 1×), 10% pyridine in EtOH (once), ethanol (2×), and Milli‐Q water (2×). The replica PEG particles were obtained by dissolution of the silica using 200 µL of buffered HF solution (13.3 M NH_4_F, 5 M HF) at pH 5.5 for 5 min at room temperature. Caution! HF is highly toxic. Extreme care should be taken when handling HF solution, and only small quantities should be prepared. The obtained replica PEG NPs were counted with Nanoparticle Tracking Analyzer (ZETAVIEW, Particle Metrix). Fluorescent labeling of the particles was done via reaction of AF488‐NHS to the hydroxyl group of HEMA. The particles were resuspended in DMSO (anhydrous, 200 µL), and AF488‐NHS (5 µL, 1 mg mL^−1^ in anhydrous DMSO) was added. Following 2 h at 20 °C under shaking, unreacted dye was removed by repeated centrifugation (10 000g, 5 min) and resuspension steps in Milli‐Q water (3×). The particles were stored in EtOH in the refrigerator (∼4 °C) prior to use. The morphology of the nanoparticles was examined using a Thermo Fisher Scientific Quattro ESEM. Samples were prepared by diluting the nanoparticle suspensions 1:1000 in ultrapure water (MQ water) and then drop‐casting 10 µL aliquots onto plasma‐cleaned silicon wafers. The dried samples were sputter‐coated with a thin layer of gold (≈10 nm thickness) and imaging was performed at an accelerating voltage of 10 kV, 3mm spot size with a working distance of 12 mm. AFM imaging was performed on similarly prepared samples using a Bruker Dimension Icon AFM in peak force tapping mode. Cantilevers with a nominal spring constant of 0.7 N m^−1^ were used, with imaging parameters set to a scan rate of 0.501 ‐ 0.8 Hz and a peak force set‐point of 720pN. Scans were acquired over 800 nm areas to visualize individual nanoparticles. Height profile analysis was conducted using Bruker NanoScope Analysis software. Transmission electron microscopy (TEM) images were obtained in a Libra120 (Carl Zeiss Microscopy Deutschland GmbH, Germany) microscope equipped with Omega‐type energy filter and operated at an acceleration voltage of 120 kV. The samples were prepared by applying 2 εL of aqueous dispersions of particles on plasma‐hydrophilised carbon/formvar coated copper grids and letting them dry at ambient conditions. Hydrodynamic diameter and polydispersity index (PDI) of the nanoparticles were measured by DLS using a Malvern Zetasizer Nano ZS instrument. Samples were prepared by adding 10 µL of nanoparticle suspension to 600 µL of ultrapure water. Measurements were performed at a scattering angle of 173° (backscatter) at 23 °C.

### Heparin Modification and in Vitro Assays

Heparin (Tinzaparin) was conjugated to the PEG‐NPs via amide coupling to the AEMA functional handle. The protecting Boc group was cleaved under acidic condition using trifluoroacetic acid (pH 2, 10 min, sonication) to yield amine functionalized NPs. Subsequently, the carboxylic acid group of heparin (1 mM) was activated with an equimolar concentration of N,N'‐dimethylmethylenediamine (DMTMM), a carbodiimide‐based coupling reagent for 15 min at room temperature. The Heparin‐DMTMM mixture was added to the amine PEG‐NPs and incubated overnight under shaking conditions at room temperature. The particles were purified of unbound heparin by subsequent washes in Milli Q water and phosphate buffered saline (PBS, pH 7.4) followed by a final resuspension in PBS. The incorporation of heparin was checked with zeta potential measurements, quantitative fluorescence analysis, CLSM‐based colocalization analysis, and FTIR. The functional aspects of heparin activity were determined by Anti‐FXa activity and plasma recalcification assessment.

For quantitative fluorescence analysis and CLSM, the heparin was labeled with AF488‐Cadaverine (cAF488), and the PEG‐NPs were labeled with N‐hydroxysuccinimide (NHS)‐activated Alexa Fluor 555 (AF555‐NHS). For cAF488 labeling of heparin, 0.42 mg N‐(3‐Dimethylaminopropyl)‐N'‐ethylcarbodiimide hydrochloride (EDC) and 0.18 mg N‐Hydroxysuccinimide (NHS) were added to 7.69 × 10⁻⁴ mmol Tinzaparin. The reaction mixture was incubated for 15 min at 5 °C. Subsequently, cAF488 (7.69 × 10⁻⁴ mmol) was added. The reaction was allowed to proceed overnight at room temperature. The heparin‐cAF488 conjugate was purified by dialysis (1000 MWCO membrane) against 800 mL of 1 m NaCl followed by three rounds of dialysis against 800 mL of water to remove any unreacted cAF488. AF555‐NHS labeling of PEG‐NPs was carried out as stated above i.e. via reaction of AF555‐NHS to the hydroxyl group of HEMA on the NPs. Heparin‐cAF488 was conjugated to PEG‐AF555 NPs as stated above via amide coupling. Successful conjugation of heparin to PEG NPs was also confirmed via FTIR (Bruker, Tensor II) by recording spectra of PEG NPs, PEG‐HEP NPs, and free heparin in wavenumber range of 400–4000 cm‐1.

For quantitative estimation of heparin, a standard curve was generated using fluorescently labeled heparin (HEP‐cAF488) to correlate fluorescence intensity with heparin concentration. The fluorescence intensity of 5 × 108 PEG‐HEP‐AF488 NPs was then interpolated onto this standard curve to determine the average heparin payload. CLSM was conducted at the appropriate excitation and emission wavelength followed by a Pearson correlation analysis to ascertain colocalization.

The anticoagulant property of heparin bound to NPs was verified as Anti‐FXa activity and plasma recalcification assay. A range of NP concentrations (1 × 10^7^, 2.5 × 10^7^, 5 × 10^7^, 7.5 × 10^7^, 1 × 10^8^) was chosen for simultaneous heparin concentration and activity quantification as a function of inhibition of Factor Xa. The ability of the PEG‐Hep NPs to inhibit Factor Xa was tested as a chromogenic assay in plasma with the Chromogenix Factor Xa Kit (Diapharma) according to the manufacturer's instructions. Briefly, the NP suspensions were centrifuged, supernatant was removed, and the NPs pellets were resuspended in PBS (37.5 µL). 12.5 uL of citrate plasma (Deutsches Rotes Kreuz) was added to the NP suspension. 250 µM of the chromogenic substrate S‐2238 (50 µL) was added to the NP‐plasma mixture and followed by addition of FXa (50 µL). Following a 2 min incubation at 37 °C and quenching the reaction with 50 µL acetic acid, the released para‐nitroaniline (pNA) was recorded at 405 nm at 37 °C using the Tecan Spark 10 M plate reader (Tecan, Switzerland). The absorbance was determined for each sample and compared to a reference without heparin (PEG‐NPs) as well as free heparin (1 U mL^−1^). Free heparin also served as the standard for estimating the heparin concentration on the NPs. To further supplement the anti‐FXa assay and assess the anticoagulant effect of the PEG‐HEP NPs, plasma clotting time was measured via turbidity measurements. 1 × 108 NPs PEG‐HEP NPs were equilibrated in PBS (50 µL) for 10 min at 37 °C. 50 µL citrate plasma was added to the NP suspension. Plasma recalcification was initiated by addition of 50 µL 25 mm CaCl2. The coagulation of plasma as a function of turbidity was recorded kinetically at 405 nm every 30 s for 90 min. The time of coagulation onset was calculated with the Magellan software (Tecan, Switzerland).

### ATIII Incorporation and anti‐FXa Assay

To decorate NPs with ATIII, PEG and PEG‐HEP NPs were resuspended in 1µm (approx. 60 µg mL^−1^) ATIII solution in PBS (10 µL) for 30 min at room temperature under shaking. Following incubation, the NPs were washed in PBS (2×, 5000g, 5 min) and resuspended in either 10 µL PBS or 100 µL PBS. ATIII decoration was done freshly before every experiment to ensure optimal ATIII activity.

The Chromogenix Factor Xa Kit (Diapharma) was adapted for the quantification of ATIII concentration on the PEG‐HEP‐ATIII NPs. The conventional method utilizes plasma as a source of ATIII to assess the heparin activity. Here the NPs served as the ATIII source to which a constant heparin amount was added, and the assay was carried out as indicated. Thereby, the standard curve was prepared in a PBS buffer system (with ATIII and heparin added as individual components) instead of plasma. The heparin concentration was fixed at 1 U mL^−1^ (corresponding to 1 × 10^8^ NPs, 50 µL), while varying concentrations of ATIII (0.005–10 ug mL^−1^, 50 µL) were added to create the curve. For the assay itself, standards or PEG‐HEP‐ATIII NPs (1 × 10^8^ NPs resuspended in 50 µL PBS) were transferred to a 96‐well plate. Then, 250 µm of the chromogenic substrate S‐2238 (50 µL) and FXa enzyme (50 µL) were added to initiate the reaction. After a 2 min incubation at 37 °C, the reaction was quenched with 50 µL acetic acid. Finally, the release of para‐nitroaniline (pNA) was measured at 405 nm and 37 °C using a Tecan Spark 10 M plate reader (Tecan, Switzerland). The ATIII concentration on the NPs was determined using Magellan software with a polynomial curve fitting of the data obtained from the standards.

### Assays with Human Blood and Derivatives

The study was approved by the ethics committee of the Sächsische Landesärztekammer under EK‐BR‐95/20‐1. Fresh venous blood was collected from healthy volunteers into either sodium heparin vacuettes (Grenier Bio‐One) or syringes pre‐loaded with 0.5 U mL^−1^ heparin (ratiopharm, Ulm). A negative CRP test (Diagnostik‐Nord, Schwerin, Germany) and a normal differential blood cell count (DxH500, Beckmann Coulter) were used as inclusion criteria. All experiments were done in triplicates and were started within 15 min of blood collection. For EDTA anticoagulated plasma isolation, 4.5 mL blood was collected and mixed with 0.5 mL 0.5M EDTA, followed by slow centrifugation (2000 g, 10 min, with brake). The supernatant was collected without disturbing the buffy coat layer and centrifuged again (8000 g, 10 min) to obtain plasma. The plasma was aliquoted into 2 mL tubes and stored at −80 °C until further use.

### Blood Assay to Measure the Association of NPs with Immune Cells and Confocal Laser Scanning Microscopy for Visualization of Association

Association assay was performed according to a previously published protocol.^[^ ^25^ ^]^ 1 × 10^6^ AF488 labeled NPs were incubated in whole blood (100 µL) for 1 h at 37 °C. RBCs were lysed by adding Pharm Lyse buffer at 40× blood volume and washed with PBS (pH 7.4, 4 mL, 2×) (800g, 5 min, 4 °C). Cells were phenotyped on ice for 1 h using antibodies against CD3 AF700 (SP34‐2 BD), CD14 APC‐H7 (MΦP9, BD), CD66b BV421 (G10F5, BD), CD56 PE (B159, BD), lineage‐1 cocktail APC (BioLegend), HLA‐DR PerCP‐Cy5.5 (G46‐6, BD), and CD19 BV650 (HIB19, BioLegend). Unbound antibodies were removed by washing and centrifugation (500g, 5 min, 4 °C) with FACS buffer. Cells were fixed in 1% w/v formaldehyde in PBS (200µL). 100 µL of the fixed samples were directly used for cell association analysis by flow cytometry (LSRFortessa, BDBiosciences, USA) and analyzed using BD FACSDiva software according previously published gating strategy.^[^ ^27^ ^]^ Remaining of the fixed samples were stained with DAPI (1 µg mL^−1^, Sigma Aldrich). The samples were then permeabilized using Triton X‐100 (0.1% in PBS) for 10 min followed by staining with AF633‐Phalloidin (Atto‐TEC, 1:200) conjugate. Between each step, the cells were washed (2×, 500 g, 5 min). For imaging, the cells were transferred onto poly‐L‐Lysine coated coverslips and imaged using Dragonfly Spinning Disc confocal microscope (Andor Technology Ltd., Belfast, UK)

### Blood Assay to Assess Hemocompatibility of NPs

1 × 10⁸ NPs, resuspended in 10 µL of PBS, were mixed with 1790 µL of whole blood, and hemocompatibility assessments were carried out as described previously.^[^ ^19^ ^]^ This blood‐NP suspension was then incubated for 2 h at 37 °C in polypropylene reaction‐tubes under continuous overhead rotation. An initial blood sample, serving as the negative control, was stained immediately while a positive control was obtained by incubating blood with 100 U mL^−1^ lipopolysaccharide at the same experimental conditions as the NPs. After 2 h incubation, the blood was analyzed with regards to CD11b expression on neutrophils and CD62P expression on platelets by flow cytometry (LSRFortessa, BD Biosciences, USA) and analyzed using BD FACSDiva software. The neutrophils were gated according to their characteristic forward and side scatter patterns. Additionally, blood samples were split and mixed with recommended anticoagulants for subsequent ELISA analysis of prothrombin fragment F1 + 2 (F1 + 2 Enzygnost, Siemens Healthineers, Germany), platelet factor 4 (DuoSet, R&D Systems) and complement factor fragment C5a (DRG Instruments GmBH, Marburg, Germany). The ELISAs were carried out according manufacturer's instructions.

### Incubation of Particles in Human Plasma for Proteomics Analysis

PEG, PEG‐HEP, and PEG‐HEP‐ATIII NPs (5 × 10⁸ particles dispersed in 10 µL PBS, triplicates) were immersed in 100 µL human EDTA anticoagulated plasma and incubated for 1 h at 37 °C under shaking conditions to allow biomolecular corona formation. Following the incubation, the particles were washed by centrifugation (10 000 g, 5min) and step‐wise resuspension in PBS (200 µL, 400 µL, 800 µL) to remove the unbound proteins. The final redispersion was transferred to a new tube, followed by a centrifugation (10 000 g, 5min) and eventual resuspension in 10 µL PBS. Proteomic analysis was performed using a combination of SDS‐PAGE, BCA assay, and mass spectrometry.

### Sodium Dodecyl Sulfate Polyacrylamide Gel Electrophoresis (SDS PAGE)

The hard corona proteins were eluted from the particles by adding NuPAGE LDS sample buffer (10 µL) and NuPAGE sample reducing agent (3 µL) to each sample and heating at 70 °C for 10 min. After dilution of the samples to 100 µL with Milli‐Q water, the particles were separated from the eluted proteins by centrifugation (12 000 g, 10 min). The supernatant was transferred to a LoBIND Eppendorf tube and loaded onto 4–15% Mini‐PROTEAN TGX Precast Protein Gels (Biorad) (30 µL per sample) to run for 50 min at 120 V till the dye front reached the end of the gel. Each gel included one lane of a standard molecular weight ladder. Gel staining was carried out using Simply Blue Safe Stain (Thermofisher Scientific). The gel images were cropped and presented with their respective standard molecular weight ladder.

### BCA Assay

Protein concentration in the hard corona of the NPs was quantified using a bicinchoninic acid (BCA) assay, following the manufacturer's protocol (Micro BCA Protein Assay Kit, Thermo Fisher Scientific). NP solutions were diluted to 150 µL with Milli‐Q water. A standard curve was generated using bovine serum albumin (BSA) at concentrations ranging from 0.5 to 2.0 mg mL^−1^. Non‐treated PEG NPs served as a negative control for blank correction. The provided working reagent (WR) was added to each standard and sample, followed by incubation at 37 °C for 2 h. After cooling to room temperature, the absorbance at 562 nm was measured using a Tecan Spark 10 M plate reader. The protein concentration on the NPs was determined using Magellan software with a best‐fit polynomial curve fitting of the data obtained from the standards.

### Mass Spectrometry

In solution tryptic digest was carried out on the biomolecular corona of the NPs for recovery of proteins, according to a previously published protocol.^[^ ^42^ ^]^ First, the 10 µL NP suspensions were topped upto 100 µL with 100mM ammonium bicarbonate buffer (NH_4_HCO_3_). The samples were reduced and alkylated in a multistep process: Reduction (2 µL, 100 mm DTT in 20mm NH_4_HCO_3_; 56°C, 30 min) followed by alkylation (5 µL, 60 mm Iodoacetamide in 20 mm NH_4_HCO_3_; RT, dark, 30 min) and another reduction (2 µL, 100 mm DTT in 20 mm NH_4_HCO_3_; RT, 15 min). After topping up to 200 µL with NH_4_HCO_3_ buffer (20 mm; pH 8.0), enzymatic digestion was carried out in solution by addition of 2 µL of MS‐grade trypsin (100 ng mL^−1^), twice every 24 h (samples were incubated at 37 °C during the digestion). This was followed by a 24 h rLys‐C digestion (2 µL, 37 °C). Digested proteins were purified using C‐18 ultra microspin columns according to the manufacturer's specifications, dried by vacuum centrifugation, and stored at −20° C until measurement. The dried samples were recovered in 50 µL of 5% formic acid in water of which 5 µL were injected for LC‐MS/MS. The peptides obtained from the respective digests were analyzed by liquid chromatography coupled with tandem mass spectrometry (LC‐MS/MS) using Q‐Exactive HF mass spectrometer (Thermo Fisher Scientific) fitted with a nanoflow HPLC (Ultimate 3000, Dionex). The spectrometer was operated in DIA mode. The MS data were processed with DIA‐NN V1.8^[^ ^43^ ^]^ and interpreted via the Perseus software.^[^ ^44^ ^]^

### Statistical Analysis

All statistical analyses were performed in Origin 2021. One‐way analysis of variance (ANOVA) was used to compare means across experimental groups, followed by Tukey's post hoc test to evaluate pairwise statistical differences among all samples. Differences were considered statistically significant at p < 0.05 or p<0.001.

## Conflict of Interest

The authors declare no conflict of interest.

## Supporting information



Supporting Information

## Data Availability

The data that support the findings of this study are available from the corresponding author upon reasonable request.
