# Supplementary material for: Coagulation Matters: ATIII‐Enriched Biomolecular Corona Enhances the Hemocompatibility of PEG Nanoparticles
Source: Adv Healthc Mater. 2025 Jun 30;14(24):2501431. doi: 10.1002/adhm.202501431 (PMC12447026; doi:10.1002/adhm.202501431)
Supplement: Supplementary file 1 — Supporting Information [file ADHM-14-0-s001.docx]

Supporting Information

Coagulation Matters: ATIII-Enriched Biomolecular Corona Enhances the Hemocompatibility of PEG Nanoparticles

Vaidehi Londhe, Manfred F. Maitz, Triantafyllos Chavakis, Carsten Werner, Alessia C. G. Weiss, Quinn A. Besford^*^

**Table S1.** Size distribution of the NP systems obtained from DLS, and as a function of time.

| NP System | Hydrodynamic diameter (nm) | | | |
| --- | --- | --- | --- | --- |
|  | **Time of synthesis** | | **One year later** | |
|  | Size (d.nm) | PDI | Size (d.nm) | PDI |
| MSNP | 87.9 ± 6 | 0.35 ± 0.02 | 80.5 ± 7 | 0.36 ± 0.20 |
| PEG | 163.4 ± 27 | 0.37 ± 0.16 | 160.8 ± 14 | 0.19 ± 0.06 |
| PEG-HEP | 337.8 ± 10 | 0.39 ± 0.02 | 358 ± 19 | 0.37 ± 0.04 |


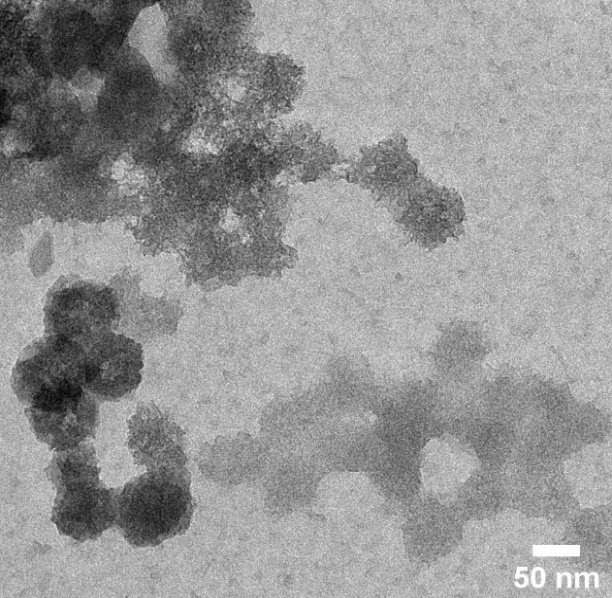


**Figure S1.** Larger scale TEM characterisation of soft replica PEG NPs.


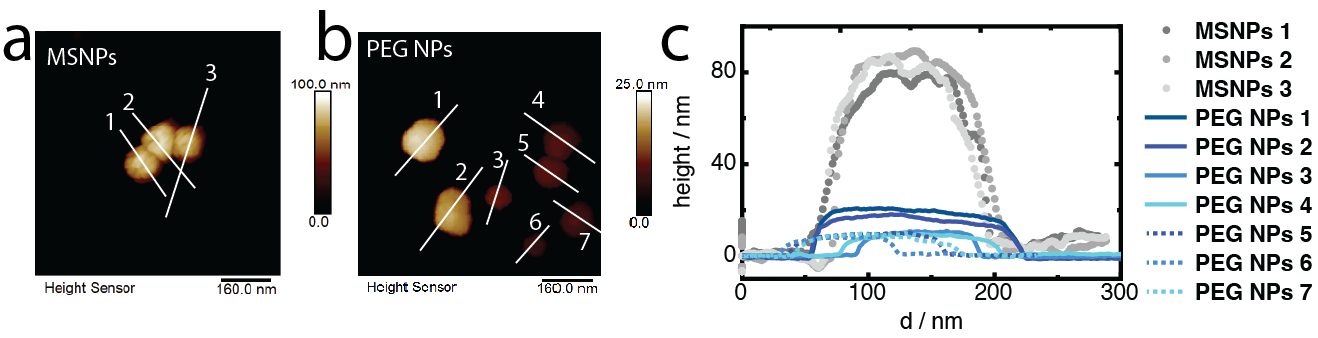


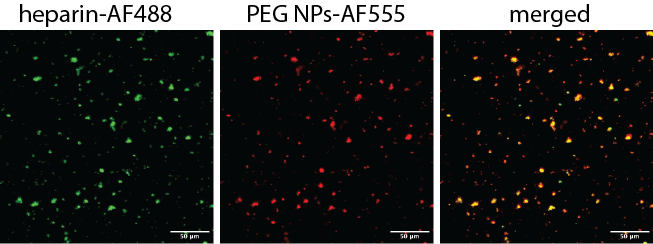
**Figure S2.** Topographic AFM images of a. MSNPs, and b. PEG NPs, with c. corresponding height profile analysis (indicated by the white lines in the respective AFM images).

**Figure S3.** Channel separated CLSM images of the PEG-HEP NPs, with heparin (AF488), PEG NPs (AF555), along with a merged image. True colocalization was determined by Pearson’s coefficient analysis (below).

**Colocalization Analysis**

Whilst the merged CLSM image hints at colocalization, in order to determine true colocalization, we performed Pearson’s correlation coefficient analysis, whereby the coefficient, *r*, was determined by

$$r= \frac{\sum\left( R_{i}-\bar{R} \right)\left( G_{i}-\bar{G} \right)}{\sqrt{\sum(R_{i}-\bar{R})^{2}\sum(G_{i}-\bar{G})^{2}}}$$

where *R_i_* and *G*_i_ corresponds to the pixel intensity on the red and green channels, respectively, and the barred quantities indicate the means on the corresponding images. This analysis revealed *r* = 0.712, indicating a significant positive relationship between the green and red channels, confirming that the heparin is conjugated to the PEG NPs.

The fluorescently-labelled heparin was further used as a basis to establish the quantity of heparin per NP. A fluorescence standard curve was established of heparin-AF488 in MQ water, at several dilutions, with excitation at 485 nm and emission collected at 535 nm. From the standard curve (Figure S3a), we could establish a linear fit of

$$I=98592 \left[ \mathrm{HEP} \right]+752.36$$

where *I* is the intensity at 535 nm, and is the heparin concentration in mg/mL, with an *R*^2^ of 0.9968. We performed triplicate measurements of PEG-HEP of known NP concentrations (from NTA measurements), from which we could calculate the amount of heparin (with molecular weight of 6,500 Da) as 43 ± 4 pg/NP. This corresponds to approximately 4.1 × 10^6^ molecules of heparin per NP. The heparin was conjugated via the AEMA monomers, which if we assume 100% conversion, would produce approximately 7.4 × 10^8^ molecules of AEMA per NP for a typical NP concentration (9.8 × 10^10^ NPs). Given that the actual conjugation of AEMA will not be 100% efficient, the concentration of heparin per NP is within the order of magnitude that we would expect.


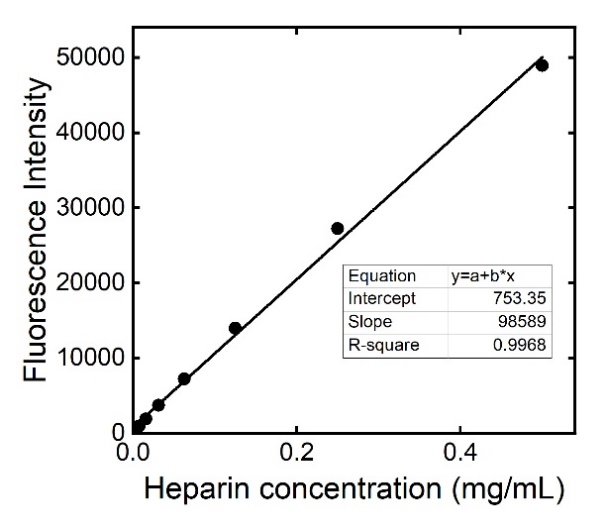


**a**

**b**


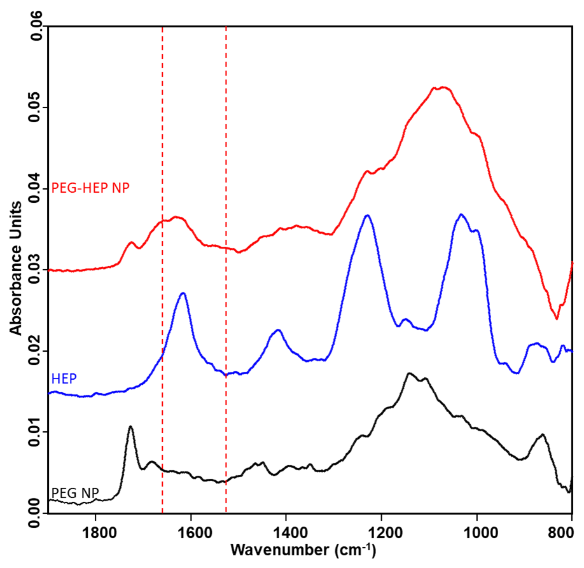


**Figure S4.** a Standard curve for the fluorescence of heparin-cAF488 as a function of concentration in MQ water. Excitation was 485 nm, and emission was collected at 535 nm. b. Fourier transform infrared spectroscopy (FTIR) analysis of PEG-HEP NPs (red), Heparin (blue) and PEG-NP (black)

**Figure S5.** Reaction scheme of Heparin conjugation and ATIII adsorption

**Figure S6.** Activity of conjugated heparin on NP (PEG-HEP) at different NP counts (absolute numbers) determined by inhibition of coagulation factor FXa against 1U/mL anti-FXa-activity of free heparin in citrated plasma, measured in a chromogenic assay.

**Figure S7**. Size distributions of ATIII based NPs before and after corona formation measured by NTA.

**b**

**a**


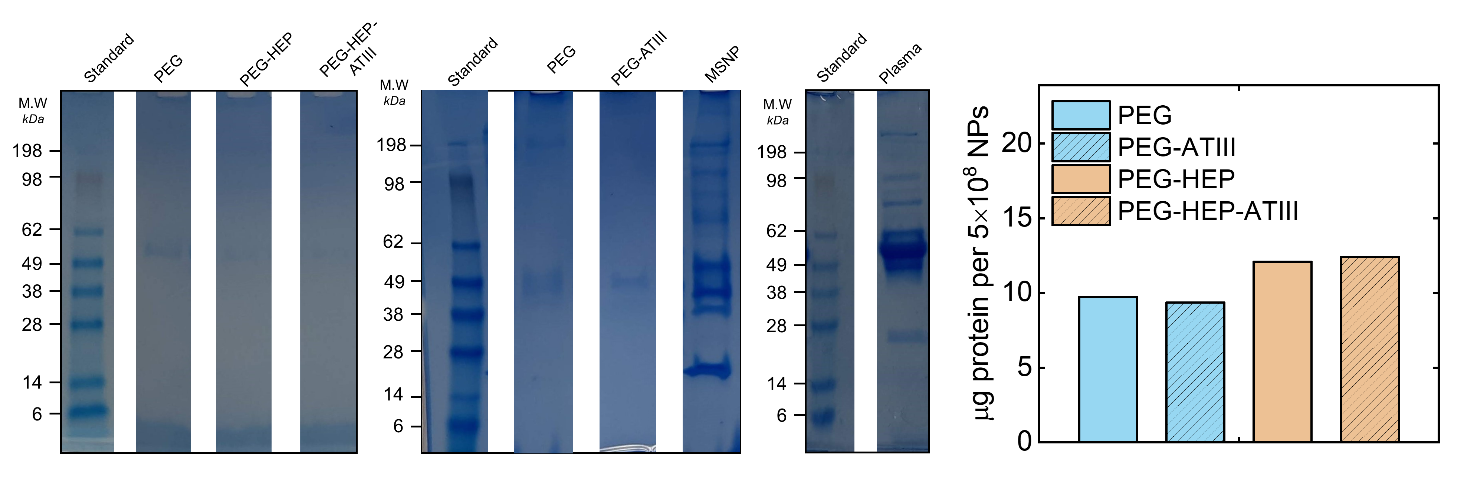


**Figure S8.** a. Characterization of hard protein corona on MSNP, PEG, PEG-ATIII, PEG-HEP, and PEG-HEP-ATIII NPs after incubation in human plasma for 1 h by SDS-PAGE analysis and b. BCA assay quantification of PEG, PEG-ATIII, PEG-HEP, and PEG-HEP-ATIII NPs

**Figure S9.** Volcano plot visualizing the differential abundance of proteins between the PEG-HEP and PEG conditions. Horizontal line marks the significance threshold (p-value = 0.01, corresponding to -log10(p-value) ≈ 2), and vertical lines at -1 and +1 indicate a 2-fold change threshold. Red points in the upper left quadrant represent features significantly less abundant in PEG-HEP compared to PEG (or significantly more abundant in PEG), while green points in the upper right quadrant represent features significantly more abundant in PEG-HEP. Gray points indicate proteins that do not meet the significance or fold change thresholds.

**Table S2**. **List of differentially abundant proteins in the protein corona from the volcano plot of PEG versus PEG-HEP nanoparticles.** The table displays protein IDs and their LFQ intensities in the PEG and PEG-HEP conditions. Proteins with red protein IDs were more abundant in the PEG corona, while those with green protein IDs were more abundant in the PEG-HEP corona. Asterisks (*) denote proteins commonly reported to influence nano-bio interactions.

| **Protein ID** | **Protein Name** | **Relative abundance by LFQ intensity** | |
| --- | --- | --- | --- |
|  |  | **PEG** | **PEG-HEP** |
| A0A075B7B8 | **Immunoglobulin heavy variable 3*** | 21.8 | 17.8 |
| A0A0B4J1U7 | **Immunoglobulin heavy variable 6-1*** | 21.3 | 19.1 |
| O75093 | Slit homolog 1 protein | 21.5 | 18.1 |
| P00734 | **Prothrombin*** | 21.8 | 20.2 |
| P00747 | **Plasminogen*** | 25.9 | 23.3 |
| P00748 | **Coagulation factor XII*** | 25.9 | 19.4 |
| P01834 | **Immunoglobulin kappa constant*** | 24.2 | 23.1 |
| P04003 | **C4b-binding protein alpha chain*** | 21.6 | 20.7 |
| P04114 | **Apolipoprotein B-100*** | 24.8 | 22.2 |
| P04196 | **Histidine-rich glycoprotein*** | 29.5 | 28.1 |
| P05155 | Plasma protease C1 inhibitor | 23.9 | 18.2 |
| P06312 | Immunoglobulin kappa variable 4-1 | 21.0 | 19.4 |
| P0C0S8 | Histone H2A.Z | 21.4 | 18.9 |
| P12259 | **Coagulation factor V*** | 23.3 | 20.1 |
| P27169 | Serum paraoxonase/arylesterase 1 | 23.9 | 22.0 |
| P35030 | Trypsin-3 | 16.7 | 18.0 |
| P35443 | **Thrombospondin-4*** | 21.1 | 17.8 |
| P48061 | Stromal cell-derived factor 1 | 16.6 | 19.3 |
| P55058 | Phospholipid transfer protein | 20.7 | 18.3 |
| Q15166 | Serum paraoxonase/lactonase 3 | 22.5 | 20.5 |
| Q92954 | Proteoglycan 4 | 22.3 | 16.6 |
| Q9BWP8 | **Collectin-11*** | 22.2 | 17.8 |
| Q9BXN1 | Asporin | 21.6 | 18.0 |
| Q9NQ79 | Cartilage acidic protein 1 | 20.4 | 18.3 |
| Q9Y6Z7 | **Collectin-10*** | 22.2 | 18.0 |
| P00746 | Complement factor D | 19.0 | 22.1 |
| P01034 | Cystatin-C | 20.9 | 22.7 |
| P01344 | Insulin-like growth factor II | 16.6 | 20.0 |
| P03950 | Angiogenin | 19.6 | 23.2 |
| P03973 | Antileukoproteinase | 15.7 | 18.9 |
| P04156 | Major prion protein | 15.9 | 17.6 |
| P22692 | Insulin-like growth factor-binding protein 4 | 20.5 | 22.1 |
| P24593 | Insulin-like growth factor-binding protein 5 | 21.7 | 24.1 |
| Q14767 | Latent-transforming growth factor beta-binding protein 2 | 19.1 | 21.5 |
| Q8TEA8 | D-aminoacyl-tRNA deacylase 1 | 15.8 | 17.1 |
| Q99969 | Retinoic acid receptor responder protein 2 | 18.4 | 21.4 |
| P35030 | Trypsin-3 | 16.7 | 18.0 |
| P48061 | Stromal cell-derived factor 1 | 16.6 | 19.3 |


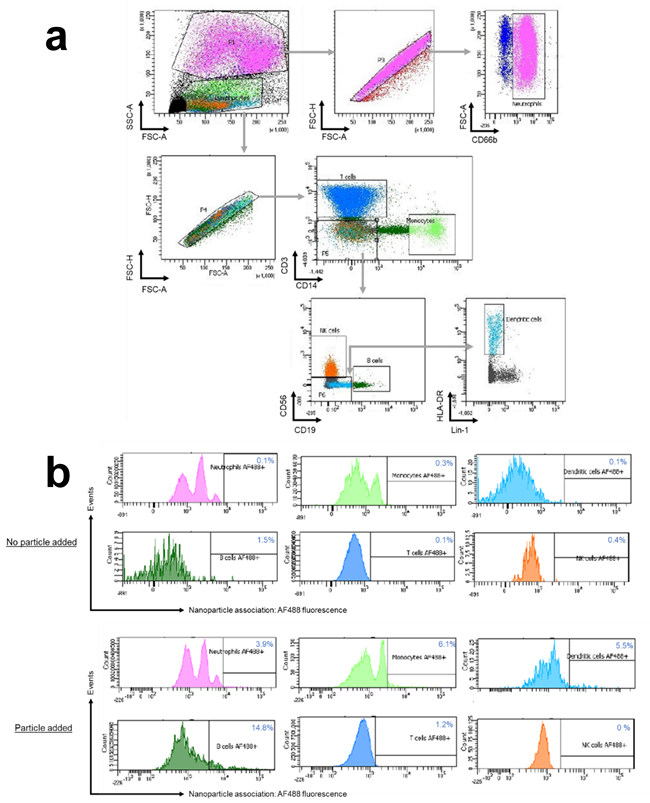


**Figure S10.** a Gating strategy used to identify white blood cell populations. Side and forward scatter were first used to locate white blood cells before doublets were excluded. The following cell types were identified based on expression of surface markers: CD66b+ neutrophils; CD3+ T cells; CD14+ monocytes; CD56+ NK cells; CD19+ B cells and Lin-, HLA-DR+ dendritic cells. b. The percentage of each cell type positive for the AF488 -NHS labeled particles was then measured and an example of the gating and particle association values for one sample is shown.

**Figure S11.** Cellular association of ATIII-decorated PEG and PEG-HEP NPs with T cells and NK cells. Data is represented as mean ± standard error; n=3. Statistical significance is determined by one-way ANOVA followed by Tukey’s test and is represented as *p<0.05.


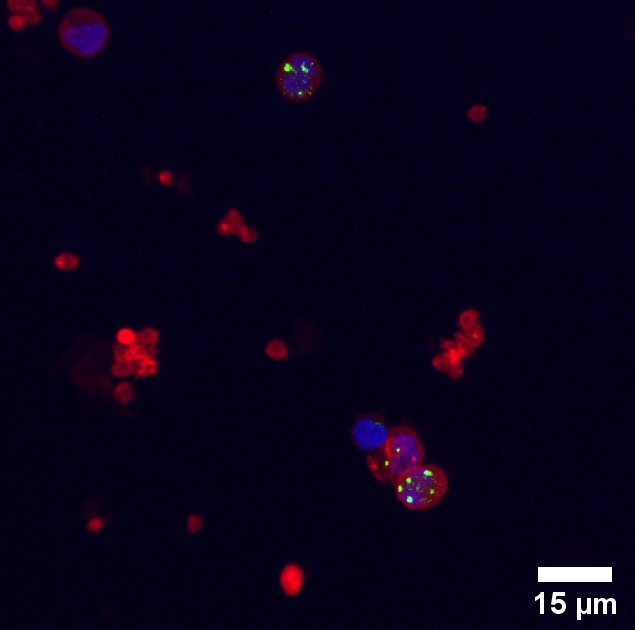


**Figure S12.** CLSM image of leukocytes following incubation of 1 × 10^6^ PEG-HEP-ATIII in 100 μL whole blood. Cell staining was performed after fixation with 1% Formaldehyde. Cell nuclei were stained with DAPI (blue). Following permeabilization, cell cytoskeleton was stained using phalloidin ATTO-633 (red). The NPs were fluorescently labelled by the attachment of AF488-NHS (green) via HEMA.


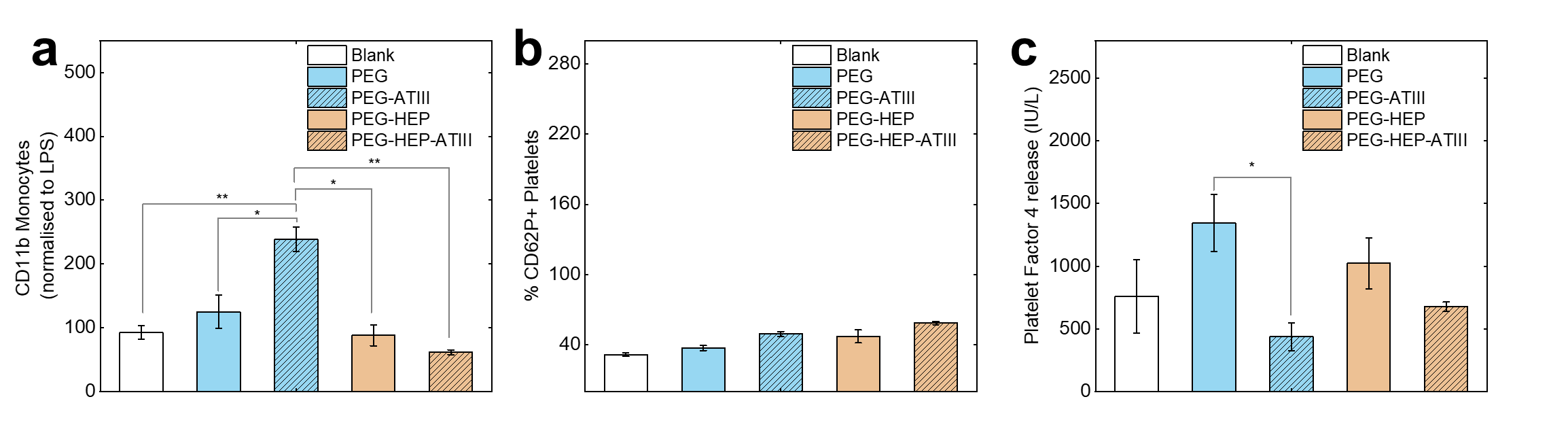


**Figure S13.** a. CD11b expression on monocytes as a marker of leukocyte inflammation (normalised to LPS). b. CD62P as a marker of platelet activation. c. Platelet factor 4 release. Data is represented as mean ± standard error; n=3. Statistical significance is determined by one-way ANOVA followed by Tukey’s test and is represented as *p<0.05, **p<0.001.
